# Maternal psychosocial risk factors and lower respiratory tract infection (LRTI) during infancy in a South African birth cohort

**DOI:** 10.1371/journal.pone.0226144

**Published:** 2019-12-30

**Authors:** Rae MacGinty, Maia Lesosky, Whitney Barnett, Polite M. Nduru, Aneesa Vanker, Dan J. Stein, Heather J. Zar

**Affiliations:** 1 Department of Paediatrics and Child Health, Red Cross War Memorial Children’s Hospital and South African Medical Research Council Unit on Child and Adolescent Health, University of Cape Town, Cape Town, South Africa; 2 Division of Epidemiology and Biostatistics, School of Public Health and Family Medicine, University of Cape Town, Cape Town, South Africa; 3 Department of Psychiatry and Mental Health, South African Medical Research Council Unit on Risk and Resilience in Mental Disorders, University of Cape Town, Cape Town, South Africa; Norwegian Institute of Public Health, NORWAY

## Abstract

**Objective:**

To investigate the association between maternal antenatal and/or postnatal psychosocial risk factors (including depression, psychological distress, alcohol abuse and intimate partner violence (IPV) and infant lower respiratory tract infection (LRTI) in a low- and middle-income-country (LMIC).

**Study design:**

Pregnant women (n = 1137) enrolled in a South African birth cohort study, the Drakenstein Child Health Study (DCHS) were longitudinally assessed for psychosocial risk factors including depression, psychological distress, alcohol abuse and/or intimate partner violence (IPV). Infants were followed from birth until one year of age for the development of LRTI by active surveillance. Two outcomes were evaluated: any LRTI, and severe and/or hospitalised LRTI. Logistic regression was used to identify associations between individual maternal psychosocial risk factors and LRTI outcomes. Analyses stratified by age were also performed to determine which age groups related to infant LRTI were linked with maternal psychosocial risk factors.

**Results:**

There were 606 LRTI episodes in 369 infants in the first year (crude incidence rate = 0.53 episodes per person-year, 95%CI: 0.50; 0.56); 31% (n = 186) of episodes were severe or hospitalised events. Maternal psychosocial risk factors were associated with LRTI and severe LRTI, particularly postnatal and long-term maternal psychological distress, antenatal maternal alcohol consumption, and postnatal maternal IPV. Age stratified analyses found that antenatal maternal alcohol consumption was associated with early infant LRTI, while antenatal maternal depression was linked with infant severe LRTI between 3 and 6 months of age, and postnatal maternal IPV was associated with early LRTI and severe forms of LRTI.

**Conclusion:**

The associations between maternal psychosocial risk factors and infant LRTI highlight the potential value of screening for maternal psychosocial risk factors in clinical settings and developing targeted interventions. Such interventions may not only improve maternal well-being, but also help reduce the burden of infant LRTI in LMIC settings.

## Introduction

Globally, lower respiratory tract infections (LRTI), particularly pneumonia, is the leading cause of death in children beyond the neonatal period, and a major cause of morbidity. LRTI accounted for approximately 920,000 deaths in children under the age of five years in 2016 [1]; most of which were preventable [2]. The burden of LRTI is highest in low and middle-income countries (LMICs), particularly in Sub-Saharan Africa and South-East Asia, in part due to the high prevalence of risk factors for LRTI, large vulnerable childhood populations and overburdened health systems [3].

Common risk factors for LRTI include tobacco smoke or indoor air pollution exposure, poor nutrition, HIV infection, low socioeconomic status, male sex, inadequate immunisation or premature birth [2, 4]. Recently, there has been growing interest in the association of maternal psychosocial risk factors, such as perinatal depression, psychological distress, substance use, and intimate partner violence (IPV), with childhood respiratory illness, including LRTI. Maternal psychosocial risk factors have been found to be associated with wheezing and asthma in early childhood [5–16]. Most studies have been conducted in high income countries (HIC), but recently we reported maternal psychosocial risk factors were associated with wheezing in early childhood in the Drakenstein Child Health Study (DCHS), a South African birth cohort in a LMIC [17]. Postnatal IPV and postnatal maternal psychological distress were found to be associated with recurrent wheezing illness in the first two years of life [17]. In addition, antenatal maternal alcohol abuse has been found to be associated with impairments in early infant lung function in the DCHS [18].

However, few studies have investigated the association between maternal psychosocial risk factors and LRTI during infancy. Antenatal or postnatal maternal depression was found to be associated with LRTI in children up until four years of age in a UK study [19]. Studies in South Asia, including Bangladesh, India and Nepal also found strong associations between postpartum maternal IPV, and early childhood infections such as diarrheal disease or respiratory tract infection [20–22]. Nevertheless, there is a paucity of research that has comprehensively evaluated the association of antenatal and postnatal maternal psychosocial risk factors with infant LRTI. There are very sparse data from Sub-Saharan Africa despite the high prevalence of both maternal psychosocial risk factors and childhood LRTI in this context [3, 23, 24].

The DCHS is a South African birth cohort study in which comprehensive measurements of maternal psychosocial risk factors have been obtained antenatally and postnatally, while infants have been closely followed for LRTI. A high prevalence of maternal psychosocial risk factors including maternal depression, psychological distress, IPV and trauma have been reported in the cohort [17, 24, 25]. In addition, a high incidence of LRTI in infancy has also been reported in this cohort, despite high immunisation coverage, adequate access to primary health care, and a low prevalence of infant HIV [3, 23].

The aim of this study was to investigate the association between maternal antenatal or postnatal psychosocial risk factors and LRTI in the first year of life, including severe LRTI, in a low resource setting in South Africa.

## Methods

Women in the 2^nd^ trimester of pregnancy were enrolled between March 2012 and March 2015 in the Drakenstein Child Health Study (DCHS), a South African birth cohort study investigating the early life determinants of child health. Mothers were then followed through childbirth and mother-child dyads continue to be followed. For this study, the follow up period was censored at one year of age. The study was located in a peri-urban area in, South Africa, in a low socioeconomic population in which more than 90% of the population access public healthcare services [3, 24].

Enrolment occurred at two antenatal clinics (Mbekweni, serving predominately a population of black African ancestry, or TC Newman, serving a mixed-ancestry population). Consenting pregnant women 18 years or older, and who intended to remain in the area for at least 1 year were enrolled [3, 26]. Infants attended study visits at 6, 10, 14 weeks, 6, 9, 12 months, and were actively followed and investigated for any LRTI episode during the first year. In addition, a follow up visit 48 hours and 4–6 weeks after the LRTI episode was conducted. For the purpose of this study, a cut-off date for each participant was considered and any LRTI episode that happened prior to this date was included. The cut-off date was either the date of early termination, the date of the 12-month scheduled visit, or the expected date of the 12-month visit if this visit was missed but the child was still active in the study.

### LRTI outcomes and surveillance

Two binary LRTI outcomes were considered in this study; any LRTI episode and severe or hospitalised LRTI, as a measure of severity, in the first year of life. These outcomes were collected through active surveillance of LRTI episodes diagnosed by trained study staff (nurses) and assessed in real time [3, 27]. LRTI was defined according to World Health Organization (WHO) criteria, which included a cough or difficulty breathing with age-appropriate tachypnoea or lower chest wall indrawing [27, 28]. Study nurses were trained in respiratory examination of children with frequent re-training [27]. Severe LRTI, defined by WHO criteria, included any general danger sign in children older than 2 months or age specific tachypnea, lower chest indrawing or general danger sign in infants less than 2 months [27, 28]. Children were hospitalised or discharged based on a treating clinician’s recommendation.

### Maternal psychosocial risk factors

Maternal psychosocial risk factor data were collected at a scheduled antenatal visit during the third trimester (between 28–32 weeks of gestation) and postnatal visits at 10 weeks, 6 months and 12 months postpartum. Several validated questionnaires, that were administered in the preferred language (English, Afrikaans or isiXhosa) of the participant, were used to measure psychosocial risk factors as has been described [24]: The Edinburgh Postnatal Depression Scale (EPDS), was used to measure maternal depression [29]. The EPDS measure asked 10 questions related to how the women felt in the previous 7 days from the time of the visit. Each of 10 questions were scored 0–3 and summed [24]. A cut-off value of 13 was used to separate the participants into above- or below-threshold for depression [29, 30]. The presence of maternal psychological distress in the past month from the time of the visit was assessed with the validated Self-Reporting Questionnaire 20-item (SRQ20) [31, 32]. Each item had a binary scoring option (0–1), and a total score was generated [24]. A cut-off value of 8 dichotomised participants into an above- or below-threshold for psychological distress [24, 33, 34].

The Intimate Partner Violence (IPV) Questionnaire was used to assess maternal physical, emotional and sexual violence exposure. The questionnaire was adapted from previous studies and measures both lifetime IPV exposure as well as recent (past 12 months) IPV exposure [35, 36]. Exposure to emotional, physical and sexual abuse were considered individually. Participants were categorised as having experienced no IPV (either emotional, physical or sexual) if all responses for that exposure were “never”; an isolated incident of IPV if one response for that exposure happened “once”; a low frequency of exposure if the response was “once” to more than one item for a particular exposure; a mid-frequency if the participant responded “a few times” to at least one item, but did not respond “many times” to any item for a particular exposure; and a high frequency if there were any responses of “many times” for a particular exposure [24]. These were further categorised into above and below threshold; where low to high frequency was considered above threshold and no exposure or an isolated incident was considered to be below threshold. A participant was determined to be above threshold for “recent” IPV exposure if the participant was above threshold for any of the three IPV sub-types in the past 12 months.

In addition, alcohol consumption during and post pregnancy were measured by the Alcohol, Smoking and Substance Involvement Screening Test (ASSIST) [37], which considers exposure of alcohol, smoking, and substance abuse in the previous 3 months from the time of the study visit. The scoring of ASSIST has been previously described [24, 37]. For this study, item responses related to frequency and timing of alcohol consumption were used to dichotomise into daily/weekly alcohol use vs no use.

Since there were 3 postnatal time points considered in this study, each of these were considered in independent regression models for maternal depression, psychological distress, and alcohol use. The 12-month visit was used to investigate postnatal IPV exposure, as the measure enquired about physical, emotional and sexual abuse by a partner in the previous 12 months. This was done to avoid any overlap between the antenatal and postnatal periods.

### Clinical and sociodemographic risk factors

Clinical and socio-demographic risk factors were longitudinally measured including child feeding practices; HIV exposure; maternal smoking and environmental tobacco smoke (ETS) exposure, assessed by self-report and urine cotinine results (from the mother collected antenatally and at birth; infant results were collected within the first year of life). Continuous cotinine values were categorised into three levels: a score less than or equal to 10 ng/mL was consider not exposed, a score greater than 10 ng/mL and less than 500 ng/mL was considered to have passive smoke exposure, and a result greater than 500 ng/mL, was recorded as active smoking [38].

Indoor air pollution (IAP) related to the child’s home environment, was measured at an antenatal (within 4 weeks of enrolment) and postnatal (between 4 and 6 months postpartum) home visit [38]. The only pollutant considered was particulate matter (PM10), as this pollutant was previously found to be associated with LRTI in the DCHS [38]. The South African National Ambient Air Quality Standards [39] were used to define expected exposure levels for each pollutant based on an averaging period of 1 year for each measure: PM10 = 40 μg/m3 [38, 39].

Birth characteristics were collected, including gestational age and birth weight, measured by study staff as previously described [40]. Birth weight/height standardised z-scores were calculated using the updated Fenton new born growth charts, which account for prematurity [41]. In addition, socio-economic status (SES) at baseline was collected, based on a composite validated score comprising four socio-economic variables: level of maternal education, employment status, household income, and asset ownership [42]. Standardised scores were divided into quartiles, which are labelled ‘low’, ‘low-moderate’, ‘high-moderate’, and ‘high’ groups [42]. The infant’s vaccination schedule was also recorded longitudinally, at scheduled visits.

### Ethical approval

The DCHS was approved by the Faculty of Health Sciences, Human Research Ethics Committee (HREC), University of Cape Town (401/2009) and by the Western Cape Provincial Health Research committee [3]. Mothers provided written informed consent at enrolment and annually thereafter.

### Statistical analysis

Analyses were conducted with STATA version 14.0 (College Station, Texas, USA). Descriptive data were presented as medians, interquartile range (IQR) and frequencies (proportions), as appropriate. Mann-Whitney rank sum and Kruskal-Wallis tests were used to test for associations between categorical and continuous variables, as all continuous variables were non-Gaussian. Pearson Chi-square test or Fisher Exact tests were used to determine if significant associations existed between categorical variables.

**Fig 1:**
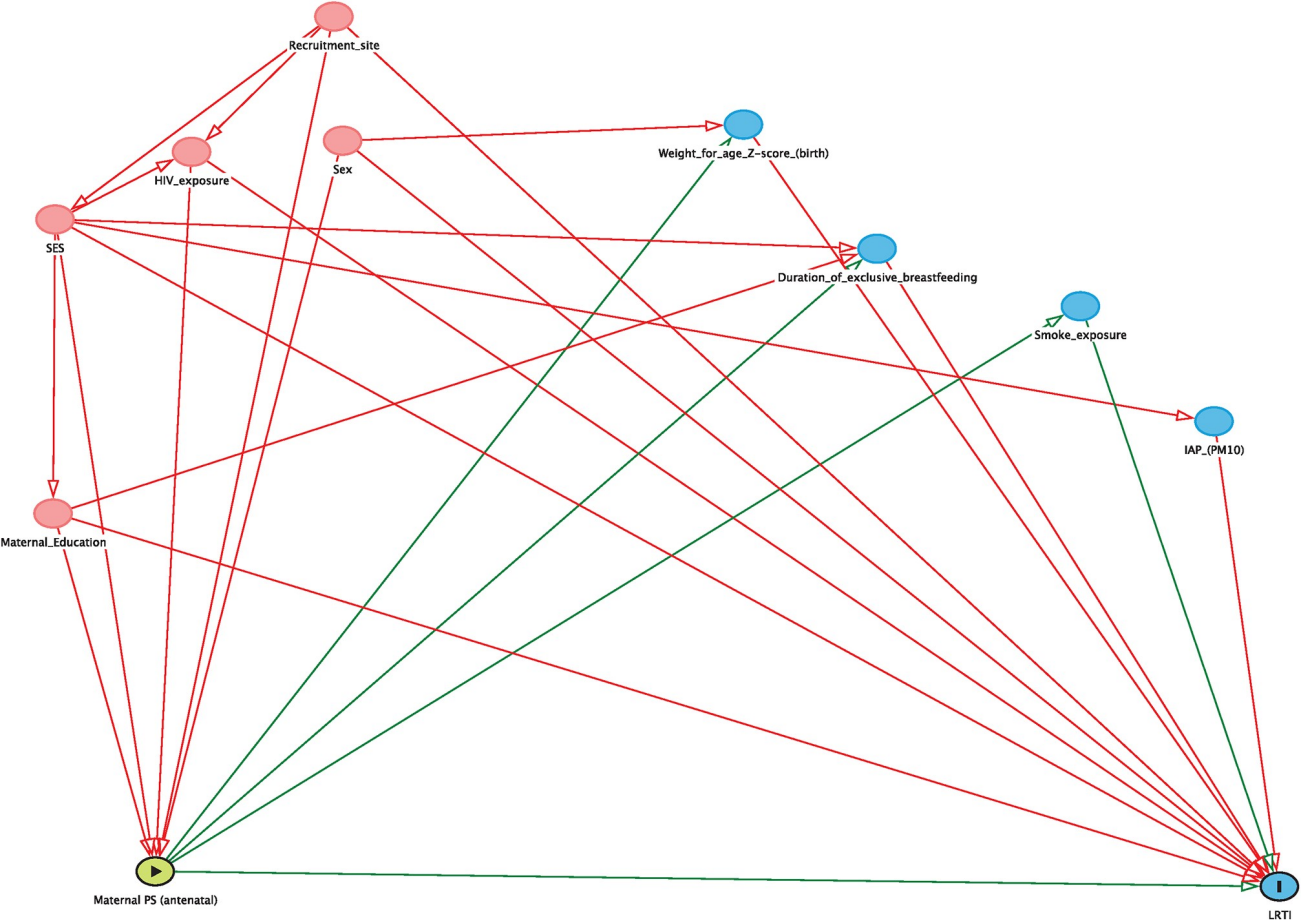
Directed acyclic graph (DAG) considering minimum set of confounding variables between antenatal maternal psychosocial risk factors and LRTI outcome.

**Fig 2:**
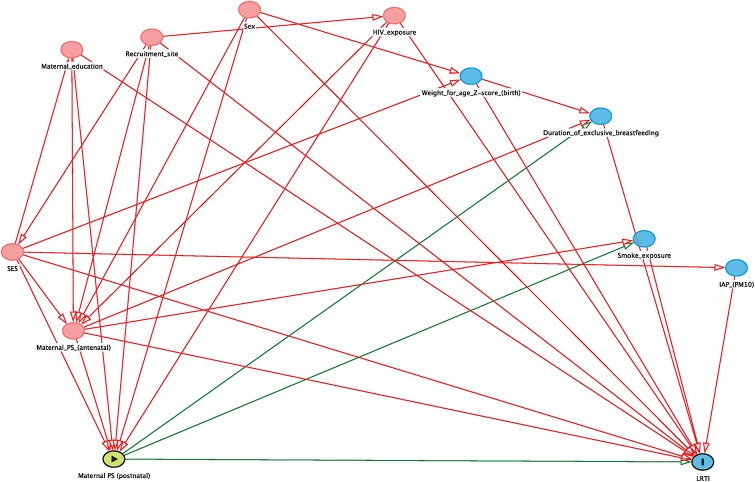
Directed acyclic graph (DAG) considering minimum set of confounding variables between postnatal maternal psychosocial risk factors and LRTI outcome.

Multiple logistic regression was used to model the association of maternal antenatal, postnatal, and long-term psychosocial risk factors with any LRTI and any severe/hospitalised LRTI episode in the first year of life adjusting for critical clinical and sociodemographic covariates. Directed acyclic graphs (DAGs) were used to identify the minimum set of confounding variables, which included sex, recruitment site, SES, maternal education achievement and HIV exposure (see Figs 1 and 2). Two sets of multiple regression models were run for each of the maternal psychosocial risk factors: the first set included the minimum set of confounder variables identified by the DAGs, and the second set included the confounding variables as well as additional variables (including mediators) based on prior literature: smoke exposure (maternal & infant urine cotinine results), indoor air pollution (assessed by PM10), weight for age z-score at birth, duration of exclusive breastfeeding and season of birth. [2, 23]. Season of birth was included to adjust for seasonality, as winter and autumn months are more commonly linked with LRTI episodes [27].

Each maternal psychosocial risk factor was considered in an individual model. In addition, antenatal and postnatal psychosocial risk factors were considered separately to estimate the association each of these exposures had on LRTI outcomes at different time points. The association of long-term psychosocial risk factor exposure on LRTI outcomes was also analysed; long-term exposure for each individual maternal psychosocial risk factor was defined as being above threshold for that psychosocial risk factor at two or more of the four time points considered in this analysis.

Since LRTI episodes are more common in earlier months of age, stratified analyses by age were also constructed [43]. Four time points were considered: 0- 3months, 3–6 months, 6–9 months and 9–12 months of age. Stratifying by age presented an opportunity to consider temporality, particularly with postnatal psychosocial risk factors, as episodes after the exposure could be analysed. This analysis also provided an opportunity to determine if the exposure took place after the outcome (reverse causation). The stratified models adjusted for sex, recruitment site, HIV exposure, maternal education achievement, SES quartile, maternal urine cotinine (smoke exposure), PM10 exposure, weight for age z-score at birth, duration of breastfeeding, season of birth and LRTI in previous age period.

Diagnostic checks were performed for all the multiple logistic regression models. These included checks for specification error, Hosmer and Lemeshow’s goodness of fit test, and checks for multicollinearity using the variance inflation factor (VIF). Influential observations were also considered using Pearson residuals, deviance residuals and leverage.

## Results

**Fig 3:**
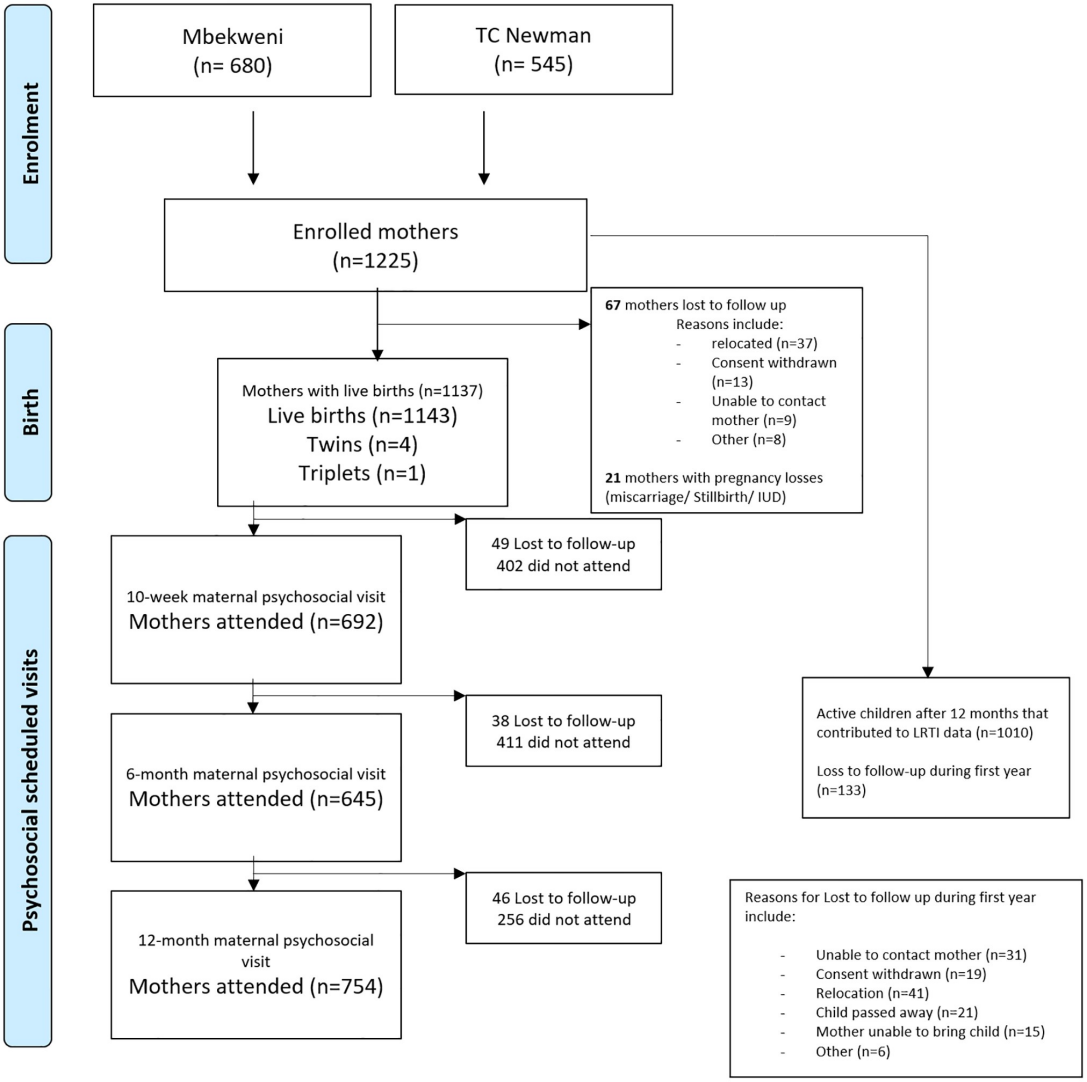
Flow diagram of attendance and loss to follow-up during the first year of life.

Among 1137 women enrolled in the study, there were 1143 live births (4 sets of twins, 1 of triplets), giving a total infant follow- up time of 1142 years in the current study. During the first year of life, 133 (12%) participants were lost to follow-up, Fig 3. Participant characteristics stratified by mothers attending or not attending antenatal or postnatal psychosocial visits, are shown in Table 1. The median maternal age was 26 (IQR 22–31) years; 22% (n = 248) of women were HIV infected, and this was not statistically difference between those attending the psychosocial visits and those not attending. Despite a high prevalence of maternal HIV, only two children were HIV-infected. Overall, based on maternal urine cotinine results, 32% of the women were classified as active smokers, with similar proportions across those attending and not attending the antenatal psychosocial visit. However, there was a significantly higher prevalence of passive and active smokers in those attending at least one psychosocial postnatal visit compared to those not attending any of the visits.

**Table 1:** Participant characteristics.

	All participants (N = 1143 children; 1137 mothers)	Participants attending antenatal visit n = 995 (87%) (1000 births from the mothers attending)	Participants not attending antenatal visit n = 142 (13%), (143 births from mothers not attending)	P-value	Participants attending at least one postnatal^a^ visit n = 980 (86%) (984 children from mother attending)	Participants not attending any postnatal visit n = 157 (14%) (159 children from mothers not attending)	P-value
**Birth & clinical factors**^b^							
Male	589 (51.5%)	519 (51.9%)	70 (48.1%)	0.509	506 (51.4%)	478 (48.6%)	0.855
Prematurity (< 37 weeks)	194 (17.0%)	159 (15.9%)	35 (24.5%)	0.011	152 (15.5%)	42 (26.4%)	0.001
Weight for age Z-score at birth, mean (SD)	-0.54 (-1.31–0.07)	-0.54 (-1.29–0.06)	-0.56 (-1.36–0.11)	0.831	-0.56 (-1.32–0.08)	-0.49 (-1.10 –-0.02)	0.662
Exclusive breastfeeding (months), mean (SD)	1.00 (0.46–3.00)	0.92 (0.46–3)	1.84 (0.57–3.21)	0.097	1.15 (0.46–3.00)	0.92 (0.23–1.84)	0.011
Season of birth							
Spring	269 (23.5%)	245 (24.5%)	24 (16.8%)	0.017	226 (23.0%)	43 (27.0%)	0.725
Summer	287 (25.1%)	256 (25.6%)	31 (21.7%)	-	250 (25.4%)	37 (23.3%)	-
Autumn	281 (24.6%)	232 (23.2%)	49 (34.3%)	-	243 (24.7%)	38 (23.9%)	-
Winter	306 (26.8%)	267 (26.7%)	39 (27.2%)	-	265 (26.9%)	41 (25.8%)	-
Recruitment site							
Mbekweni	634 (55.6%)	545 (54.5%)	89 (62.2%)	0.082	536 (54.5%)	98 (61.6%)	0.092
TC Newman	509 (44.4%)	455 (45.5%)	54 (37.8%)	-	448 (45.5%)	61 (38.4%)	-
**Maternal or household factors**^c^							
Maternal age at enrolment (years)	25.76 (21.98–30.82)	25.76 (21.89–30.82)	25.73 (22.45–30.49)	0.760	25.89 (22.00–30.84)	24.77 (21.96–30.03)	0.204
Maternal HIV	248 (21.7%)	217 (21.7%)	31 (21.7%)	0.905	221 (22.5%)	27 (17.2%)	0.136
Antenatal maternal smoking^d^							
Non- smoker	262/1088 (24.2%)	230/983 (23.0%)	32/105 (22.4%),	0.266	215/952 (22.6%)	47/136 (34.6%)	0.009
Passive smoker	474/1088 (43.6%)	433/983 (44.1%)	41/105 (39.1%)	-	425/952 (44.6%)	49/136 (36.0%)	-
Active smoker	352/1088 (32.4%)	320/983 (32.6%)	32/105 (30.5%)	-	312/952 (32.8%)	40/136 (29.4%)	-
Infant smoke exposure^b^							
No smoke exposure	281/868 (32.4%)	248/774 (32.0%)	33/94 (35.1%),	0.631	246/787 (31.3%)	35/81 (43.2%)	0.091
Passive smoke exposure	469/868 (54.0%)	418/774 (54.0%)	51/94 (54.3%)	-	432/787 (54.9%)	37/81 (45.7%)	-
Active smoke exposure	118/868 (13.6%)	108/774 (14.0%)	10/94 (10.6%)	-	109/787 (13.9%)	9/81 (11.1%)	-
Indoor air pollution: PM10 ^d^	140/767 (18.3%)	120/694 (17.3%)	20/73 (27.4%)	0.033			
Maternal educational achievement							
Primary	86 (7.6%)	81 (8.1%)	5 (3.5%)	0.044	74 (7.6%)	12 (7.6%)	0.091
Some secondary	606 (53.3%)	533 (53.6%)	73 (51.4%)	-	536 (54.7%)	70 (44.6%)	-
Completed secondary	372 (32.7%)	323 (32.5%)	49 (34.5%)	-	311 (31.7%)	61 (38.9%)	-
Any tertiary	73 (6.4%)	58 (5.8%)	15 (10.6%)	-	59 (6.0%)	14 (8.9%)	-
Household size							
1–3 people	379 (33.4%)	334 (33.6%)	45 (31.7%)	0.316	322 (32.9%)	57 (36.3%)	0.641
4–5 people	386 (34.0%)	330 (33.2%)	56 (39.4%)	-	337 (34.4%)	49 (31.2%)	-
More than 6 people	372 (32.7%)	331 (33.3%)	41 (28.9%)	-	321 (32.8%)	51 (32.5%)	-
Monthly Household income							
<1000 [ZAR]	430 (37.8%)	384 (38.6%)	46 (32.4%)	0.203	384 (31.2%)	46 (29.3%)	0.054
1000–5000 [ZAR]	553 (48.6%)	474 (47.6%)	79 (55.6%)	-	468 (47.8%)	85 (54.1%)	-
>5000 [ZAR]	154 (13.6%)	137 (13.8%)	17 (12.0%)	-	128 (13.1%)	26 (16.6%)	-
Socioeconomic status quartile							
Lowest	274 (24.1%)	244 (24.5%)	30 (21.1%)	0.205	239 (24.4%)	35 (22.3%)	0.013
Low—moderate	293 (25.8%)	263 (26.4%)	30 (21.1%)	-	264 (26.9%)	29 (18.5%)	-
High—moderate	288 (25.3%)	250 (25.1%)	38 (26.8%)	-	249 (25.4%)	39 (24.8%)	-
Highest	282 (24.8%)	238 (23.9%)	44 (31.0%)	-	228 (23.3%)	54 (34.4%)	-

SD, standard deviation; HIV, Human Immunodeficiency Virus; PM10, particulate matter, between 2.5 and 10 microns in diameter. Note: Infant vaccinations: Pneumococcal Conjugate Vaccine (PCV13) administration: 99.6% (n = 983/987) received at 6–10 weeks; 99.3% (n = 945/952) received at 14 weeks; 98.5% (n = 868/881) received at 9 months. Diphtheria, Tetanus, Acellular Pertussis, Polio and Haemophilus Influenzae type b (DTaP-IPV-Hib) vaccination administration: 99.5% (n = 981/986) received at 6 weeks; 99.5% (n = 967/972) received at 10 weeks; 99.2% (n-943/952) received at 14 weeks.

^a^ postnatal attendance based on attending atleast one psychosocial visit (visits took place at 10 weeks, 6 months and 12 months post-delivery).

^b^ Based on 1143 live births.

^c^ based on 1137 mothers recruited.

^d^ Missing data.

Approximately 87% of participants lived in households that earned less than 5000 South African Rand (approximately 350 USD) a month, and the level of household income was not statistically different between those attending and not attending psychosocial visits, Table 1. Most women had not completed high school (n = 606, 53%).

Most children were born healthy; the median (IQR) birth weight was 3.1 (2.7–3.4) kg, while 194 (17%) of births were premature (<37 weeks’ gestation), predominantly late premature. The mean duration of exclusive breastfeeding was only 1.9 months, however vaccination coverage was very high, as approximately 99% of vaccinations were administered at 6–10, 14 weeks and 9 months scheduled visits, Table 1.

### Maternal psychosocial risk factors

Antenatal depression was present in 237 (24%) of women; while 20% (n = 201) of the women suffered from antenatal psychological distress, with majority in the women attending TC Newman (Table 2). A high prevalence of antenatal IPV was also observed in the cohort, with 334 (34%) of participants exposed in the past year. In addition, 45 (4.5%) of the women in the cohort consumed alcohol either daily or weekly during pregnancy.

**Table 2:** Maternal psychosocial risk factors & infant LRTI episodes in the first year of life.

	All participants	Participants from Mbekweni	Participants from TC Newman	P-value
***Maternal psychosocial risk factors***^a^				
***Antenatal (n = 995)***				
Depression	237 (23.6%)	124/540 (23.0%)	113/454 (24.9%)	0.48
Psychological distress	201 (20.2%)	92/538 (17.1%)	109/453 (24.1%)	0.01
IPV (recent)^b^	334 (33.6%)	150/539 (27.8%)	184/455 (40.4%)	<0.001
Alcohol daily/weekly use	45 (4.5%)	10 (1.9%)	35 (7.7%)	<0.001
***Postnatal at 10 weeks (n = 692)***				
Depression	118 (17.1%)	61/367 (16.6%)	57/325 (17.5%)	0.761
Psychological distress	69 (10.0%)	18/367 (4.9%)	51 /325 (15.7%)	<0.001
IPV (Recent)	177 (25.6%)	78/367 (21.3%)	99/325 (30.5%)	0.006
Alcohol daily/weekly use	27 (3.9%)	6/367 (1.6%)	21/325 (6.5%)	0.001
***Postnatal at 6 months (n = 645)***				
Depression	97 (15.0%)	28/326 (8.6%)	69/319 (21.6%)	<0.001
Psychological distress	61 (9.5%)	5/326 (1.5%)	56/319 (17.6%)	<0.001
IPV (Recent)	184 (28.5%)	69/326 (21.1%)	115/319 (36.1%)	<0.001
Alcohol daily/weekly use	37 (5.7%)	9/326 (2.7%)	28/319 (8.8%)	0.001
***Postnatal at 12 months***				
Depression	115/717 (16.0%)	35/376 (9.3%)	80/341 (23.5%)	<0.001
Psychological distress	70/754 (9.3%)	11/399 (2.8%)	59/355 (16.6%)	<0.001
IPV (Recent)	199/742 (26.8%)	68/388 (17.5%)	131/354 (37.0%)	<0.001
Alcohol daily/weekly use	62/640 (9.7%)	17/321 (5.3%)	45/319 (14.1%)	<0.001
Infant LRTI episodes**				
Total LRTI episodes^c^^,^ ^d^^,^^e^^,^^f^	606 (100%)	374 (61.7%)	232 (38.3%)	
Severe episodes^g^^,^^h^	107 (17.7%)	70 (65.4%)	37 (34.6%)	
Hospitalised episodes^h^^,^^i^	134 (22.1%)	72 (53.7%)	62 (46.3%)	
Children with recurrent episodes (>2)	139 (12.1%)	90 (14.2%)	49 (9.6%)	
Season of LRTI				
Spring	163 (26.9%)	98 (60.1%)	65 (39.9%)	
Summer	83 (13.7%)	53 (63.9%)	30 (36.1%)	
Autumn	154 (25.4%)	109 (70.8%)	45 (29.2%)	
Winter	206 (34.0%)	114 (55.3%)	92 (44.7%)	

IPV, Intimate Partner Violence

^a^ Long-term maternal psychosocial exposure (above threshold at 2 or more of the scheduled psychosocial visits): 120 (10.5%) women exposed to long-term IPV; 84 (7.4%) women with long-term psychological distress; 131 (11.5%) women with long-term exposure to depressive symptoms; 25 (2.2%) women with long-term alcohol use

^b^ IPV antenatal lifetime exposure N = 453 (46%); recent exposure = past 12 months

^c^ *LRTI episodes over time*: *0–3 months*: *N = 163; 3–6 months*: *N = 211; 6–9 months*: *N = 118; 9–12 months*: *N = 114 (24 of these episodes took place after the child was 12 months* old but before the 12-month schedule visit was attended)

^d^ Crude incidence rate = 530.65 per 1000 person years (95% CI: 501.22; 559.92

^e^ Crude incidence rate by recruitment site: African-ancestry population = 592.44 per 1000 per years (95% CI: 553.22; 631.33); mixed-ancestry population = 453.82 per 1000-person years (95% CI: 410.24; 498.32)

^f^ Number of infants with at least one LRTI episode: 369 (215 infants from African-ancestry population; 154 infants from mixed-ancestry population)

^g^ Number of infants with at least one severe LRTI episode: 98

^h^ **Severe and hospitalised episodes = 186** (113 from African-ancestry population, 73 from mixed-ancestry population); number of infants with at least on severe and/or hospitalised episode: 152

^i^ Number of infants with at least one hospitalised LRTI episode: 109

** P-values excluded as this is a description of total events in the cohort

Postnatal psychosocial risk factors were considerably more prevalent in the women attending TC Newman compared to those attending Mbekweni, particularly in the case of psychological distress and IPV. Postnatal IPV was highly prevalent in the cohort, with 199/742 (27%) exposed postnatally. In addition, 118/692 women (17%), 97/645 (15%), and 115/717 (16%) were considered to be above threshold for postnatal depressive symptoms at the 10-week, 6-month and 12-month scheduled visits, respectively.

In terms of long-term psychosocial exposures, a total of 131 women (12%) were found to display depressive symptoms longitudinally, 84 (7%) women suffered from long-term psychological distress, and 120 women (11%) were exposed to long term IPV (Table 2).

### LRTI episodes

There were 606 episodes of LRTI which occurred in the cohort from 369 children (crude incidence rate of 0.53 episodes per person year, 95% CI: 0.50; 0.56) over the first year. Most episodes occurred in the first 6 months of life (n = 374; 62%), with 163 episodes in the first 3 months and 211 episodes between 3 and 6 months. Furthermore, 118 episodes occurred between 6 and 9 months, and 114 episodes were identified between 9 and 12 months (this includes 24 episodes that took place after the child was 12 months but before the child attended the 12-month scheduled visit). In addition, 107 (18%) of the episodes were classified as severe. There were 139 (12%) children who had recurrent episodes during the first year of life. Hospitalisation occurred in 134 (22%) of episodes (Table 2). Further, 186 (31%) episodes were either classified as severe or the child was hospitalised.

Infants from the Mbekweni clinic experienced a higher proportion of LRTI (62%; crude incidence rate of 0.59 per person year, 95% CI: 0.55; 0.63), relative to infants from TC Newman (0.45 per person year, 95% CI: 0.41; 0.50).

### Antenatal maternal psychosocial risk factors associations with LRTI

#### Antenatal depression

Antenatal maternal depression was not associated with either any LRTI or severe LRTI in the first year of life, Table 3–5. However, antenatal maternal depression was associated with severe LRTI between 3 and 6 months of age when the minimum set of confounder variables were included in the model (adjusted OR = 2.55, 95% CI: 1.08; 6.01, p-value = 0.032), Table 6. However, this association disappeared when mediator variables such as maternal smoking and indoor air pollution were included, S2 Table.

**Table 3:** Logistic regression—LRTI ever vs maternal psychosocial risk factors.

Maternal psychosocial risk factor	Unadjusted OR (95% CI), p-value	Adjusted^a^ OR (95% CI), p-value	Adjusted^b^ OR (95% CI), p-value
***Depression***			
*Antenatal* (n adjusted 1 = 994; n adjusted 2 = 651)	1.13 (0.83; 1.53), 0.451	1.06 (0.77; 1.46), 0.716	0.86 (0.58; 1.28), 0.456
*Postnatal– 10 weeks* (n adjusted 1 = 627; n adjusted 2 = 355)	0.91 (0.59; 1.41), 0.681	0.77 (0.47; 1.26), 0.298	1.18 (0.61; 2.27), 0.624
*Postnatal– 6 months* (n adjusted 1 = 579; n adjusted 2 = 325)	1.05 (0.67; 1.65), 0.832	1.17 (0.70; 1.94), 0.554	1.21 (0.63; 2.31), 0.569
*Postnatal– 12 months* (n adjusted 1 = 653; n adjusted 2 = 366)	1.32 (0.88; 1.98), 0.183	1.54 (0.98; 2.43), 0.064	**2.06 (1.08; 3.96), 0.029**
*Long-term exposure* (n adjusted 1 = 1140; n adjusted 2 = 705)	1.21 (0.82; 1.77), 0.332	1.12 (0.75; 1.68), 0.566	1.05 (0.66; 1.67), 0.840
***Psychological distress***			
*Antenatal* (n adjusted 1 = 991; n adjusted 2 = 651)	1.18 (0.86; 1.64), 0.306	1.19 (0.85; 1.66), 0.312	1.44 (0.95; 2.17), 0.085
*Postnatal– 10 weeks* (n adjusted 1 = 628; n adjusted 2 = 355)	1.03 (0.61; 1.76), 0.906	1.14 (0.63; 2.05), 0.670	2.25 (0.96; 5.27), 0.063
*Postnatal– 6 months* (n adjusted 1 = 578; n adjusted 2 = 325)	1.20 (0.70; 2.07), 0.510	1.18 (0.62; 2.23), 0.618	0.93 (0.37; 2.35), 0.872
*Postnatal– 12 months* (n adjusted 1 = 684; n adjusted 2 = 384)	1.18 (0.72; 1.95), 0.511	1.16 (0.66; 2.05), 0.603	1.21 (0.56; 2.66), 0.627
*Long-term exposure* (n adjusted 1 = 1140; n adjusted 2 = 705)	1.38 (0.87; 2.18), 0.172	1.52 (0.94; 2.47), 0.090	1.46 (0.81; 2.63), 0.212
***IPV***^**3**^			
*Antenatal* (n adjusted 1 = 994; n adjusted 2 = 651)	1.06 (0.80; 1.40), 0.689	1.05 (0.79; 1.41), 0.721	1.00 (0.69; 1.45), 0.997
*Postnatal– 12 months* (n adjusted 1 = 675; n adjusted 2 = 381)	**1.42 (1.02; 1.98), 0.038**	**1.50 (1.03; 2.18), 0.032**	1.46 (0.88; 2.43), 0.147
*Long-term exposure* (n adjusted 1 = 1140; n adjusted 2 = 705)	1.35 (0.91; 2.01), 0.130	1.40 (0.92; 2.12), 0.113	1.39 (0.85; 2.29), 0.193
***Alcohol exposure***			
*Antenatal* (n adjusted 1 = 993; n adjusted 2 = 650)	1.53 (0.84; 2.81), 0.168	1.61 (0.86; 3.02), 0.139	1.36 (0.61; 3.03), 0.449
*Postnatal– 10 weeks* (n adjusted 1 = 625; n adjusted 2 = 354)	1.31 (0.59; 2.91), 0.506	0.65 (0.26; 1.63), 0.362	0.85 (0.23; 3.05), 0.797
*Postnatal– 6 months* (n adjusted 1 = 571; n adjusted 2 = 323)	0.81 (0.39; 1.68), 0.577	0.86 (0.40; 1.83), 0.691	1.14 (0.40; 3.20), 0.806
*Postnatal– 12 months* (n adjusted 1 = 583; n adjusted 2 = 332)	1.48 (0.88; 2.51), 0.142	1.15 (0.64; 2.08), 0.640	1.54 (0.65; 3.67), 0.327
*Long-term exposure* (n adjusted 1 = 1140; n adjusted 2 = 705)	1.74 (0.78; 3.87), 0.175	1.88 (0.83; 4.29), 0.132	1.61 (0.61; 4.26), 0.339

IPV, Intimate partner violence

^a^ Adjusted based on directed acyclic graph (DAG)–adjusted for antenatal maternal psychosocial risk factor (in postnatal models); sex; recruitment site; HIV exposure; maternal education achievement; SES quartile

^b^ Adjusted based on DAG with additional variables–adjusted for antenatal maternal psychosocial risk factor (in postnatal models); sex; recruitment site; HIV exposure; maternal education achievement; SES quartile; maternal urine cotinine (smoke exposure); PM10; weight for age z-score at birth; duration of breastfeeding; season of birth

**Table 4:** Logistic regression—LRTI ever vs maternal psychosocial risk factors stratified by age.

	0–3 months^a^	3–6 months^b^	6–9 months^c^	9–12 months^d^
Maternal psychosocial risk factor	Adjusted OR (95%CI), p-value	Adjusted OR (95%CI), p-value	Adjusted OR (95%CI), p-value	Adjusted OR (95%CI), p-value
***Depression***				
*Antenatal* (n 1 = 994; n 2 = 994; n 3 = 994; n 4 = 994)	1.26 (0.81; 1.94), 0.301	1.26 (0.85; 1.89), 0.255	0.89 (0.53; 1.51), 0.674	0.69 (0.39; 1.21), 0.197
*Postnatal—10 weeks* (n 1 = 627; n 2 = 627; n 3 = 627; n 4 = 627)	1.26 (0.66; 2.39), 0.479	0.71 (0.36; 1.41), 0.329	1.14 (0.54; 2.43), 0.725	0.89 (0.39; 2.05), 0.787
*Postnatal—6 months* (n 1 = 579; n 2 = 546; n 3 = 579; n 4 = 579)	1.65 (0.86; 3.18), 0.134	0.70 (0.34; 1.43), 0.328	1.25 (0.54; 2.92), 0.605	0.91 (0.39; 2.12), 0.826
*Postnatal—12 months* (n 1 = 653; n 2 = 653; n 3 = 653; n 4 = 653)	**1.97 (1.11; 3.47), 0.020**	1.40 (0.80; 2.44), 0.237	0.83 (0.40; 1.72), 0.614	1.08 (0.54; 2.13), 0.835
***Psychological distress***				
*Antenatal* (n 1 = 991; n 2 = 991; n 3 = 991; n 4 = 991)	1.22 (0.77; 1.94), 0.389	1.48 (0.98; 2.23), 0.065	1.08 (0.62; 1.88), 0.777	0.91 (0.52; 1.58), 0.738
*Postnatal—10 weeks* (n 1 = 628; n 2 = 628; n 3 = 628; n 4 = 628)	1.63 (0.76; 3.43), 0.211	1.25 (0.57; 2.75), 0.579	2.12 (0.92; 4.89), 0.079	0.73 (0.24; 2.23), 0.576
*Postnatal—6 months* (n 1 = 578; n 2 = 546; n 3 = 578; n 4 = 578)	**2.50 (1.11; 5.61), 0.026**	0.81 (0.35; 1.87), 0.618	0.98 (0.33; 2.87), 0.969	1.36 (0.51; 3.64), 0.545
*Postnatal—12 months* (n 1 = 684; n 2 = 684; n 3 = 684; n 4 = 684)	1.65 (0.79; 3.45), 0.181	1.13 (0.57; 2.24), 0.735	1.81 (0.79; 4.13), 0.159	0.66 (0.24; 1.82), 0.423
***IPV***^***5***^				
*Antenatal* (n 1 = 994; n 2 = 994; n 3 = 994; n 4 = 994)	0.97 (0.64; 1.46), 0.890	1.35 (0.93; 1.96), 0.110	1.18 (0.73; 1.91), 0.499	1.13 (0.71; 1.78), 0.612
*Postnatal—10 weeks* (n 1 = 628; n 2 = 628; n 3 = 628; n 4 = 628)	1.44 (0.83; 2.51), 0.194	1.21 (0.71; 2.09), 0.484	0.86 (0.43; 1.71), 0.662	1.24 (0.65; 2.36), 0.513
*Postnatal—6 months* (n 1 = 577; n 2 = 544; n 3 = 577; n 4 = 577)	2.89 (1.62; 5.16), <0.001*	1.44 (0.83; 2.52), 0.195	1.05 (0.52; 2.12), 0.900	1.10 (0.58; 2.10), 0.761
*Postnatal—12 months* (n 1 = 675; n 2 = 675; n 3 = 644; n 4 = 675)	2.13 (1.30; 3.48), 0.003*	0.93 (0.58; 1.50), 0.770	0.90 (0.49; 1.63), 0.720	0.99 (0.57; 1.73), 0.971
***Alcohol exposure***				
*Antenatal* (n 1 = 993; n 2 = 993; n 3 = 993; n 4 = 993)	2.90 (1.40; 6.03), 0.004*	0.49 (0.18; 1.33), 0.162	**4.21 (1.92; 9.23), <0.001**	1.35 (0.50; 3.66), 0.555
*Postnatal—10 weeks* (n 1 = 625; n 2 = 625; n 3 = 625; n 4 = 625)	0.46 (0.12; 1.74), 0.254	0.81 (0.22; 2.92), 0.747	2.37 (0.72; 7.84), 0.156	0.59 (0.12; 2.90), 0.516
*Postnatal—6 months* (n 1 = 571; n 2 = 538; n 3 = 571; n 4 = 571)	1.25 (0.44; 3.53), 0.668	1.01 (0.39; 2.60), 0.977	0.72 (0.19; 2.75), 0.627	0.45 (0.10; 2.03), 0.301
*Postnatal—12 months* (n 1 = 583; n 2 = 583; n 3 = 583; n 4 = 583)	1.39 (0.66; 2.94), 0.386	1.33 (0.67; 2.66), 0.417	0.43 (0.16; 1.21), 0.110	1.09 (0.46; 2.56), 0.841

IPV, Intimate partner violence.

^a-d^ Multiple logistic regression models adjusted for antenatal maternal psychosocial risk factor (in postnatal models); sex; recruitment site; HIV exposure; maternal education achievement; SES quartile; and LRTI in previous period

* Still significant if 1% significance level considered

**Table 5:** Logistic regression—Severe/hospitalized LRTI vs maternal psychosocial risk factors.

Maternal psychosocial risk factor	Unadjusted OR (95% CI), p-value	Adjusted^a^ OR (95% CI), p-value	Adjusted^b^ OR (95% CI), p-value
***Depression***			
*Antenatal* (n adjusted 1 = 994; n adjusted 2 = 651)	1.27 (0.84; 1.92), 0.248	1.24 (0.82; 1.89), 0.311	0.99 (0.57; 1.72), 0.964
*Postnatal– 10 weeks* (n adjusted 1 = 627; n adjusted 2 = 355)	1.27 (0.74; 2.20), 0.386	1.12 (0.60; 2.10), 0.712	**2.24 (1.02; 4.93), 0.045**
*Postnatal– 6 months* (n adjusted 1 = 579; n adjusted 2 = 325)	1.05 (0.57; 1.94), 0.882	1.16 (0.59; 2.28), 0.675	1.46 (0.64; 3.33), 0.367
*Postnatal– 12 months* (n adjusted 1 = 653; n adjusted 2 = 366)	1.36 (0.80; 2.30), 0.253	1.58 (0.88; 2.83), 0.129	1.62 (0.74; 3.54), 0.229
*Long-term exposure* (n adjusted 1 = 1140; n adjusted 2 = 705)	1.20 (0.72; 2.00), 0.481	1.23 (0.73; 2.08), 0.442	1.17 (0.62; 2.22), 0.631
***Psychological distress***			
*Antenatal* (n adjusted 1 = 991; n adjusted 2 = 651)	1.29 (0.84; 1.99), 0.245	1.35 (0.87; 2.09), 0.184	1.43 (0.81; 2.51), 0.215
*Postnatal– 10 weeks* (n adjusted 1 = 628; n adjusted 2 = 355)	1.72 (0.92; 3.24), 0.091	2.00 (0.99; 4.05), 0.055	4.38 (1.66; 11.61), 0.003*
*Postnatal– 6 months* (n adjusted 1 = 578; n adjusted 2 = 325)	1.40 (0.70; 2.81), 0.342	1.75 (0.76; 4.00), 0.186	2.43 (0.79; 7.47), 0.121
*Postnatal– 12 months* (n adjusted 1 = 684; n adjusted 2 = 384)	1.35 (0.71; 2.55), 0.360	1.41 (0.67; 2.97), 0.361	2.27 (0.88; 5.91), 0.092
*Long-term exposure* (n adjusted 1 = 1140; n adjusted 2 = 705)	2.04 (1.18; 3.50), 0.010*	2.60 (1.45; 4.65), 0.001*	3.03 (1.48; 6.21), 0.003*
***IPV***^**3**^			
*Antenatal* (n adjusted 1 = 994; n adjusted 2 = 651)	0.97 (0.66; 1.43), 0.892	1.02 (0.69; 1.52), 0.925	1.08 (0.64; 1.82), 0.771
*Postnatal– 12 months* (n adjusted 1 = 675; n adjusted 2 = 381)	**1.70 (1.11; 2.61), 0.015**	2.02 (1.25; 3.28), 0.004*	**1.93 (1.00; 3.70), 0.049**
*Long-term exposure* (n adjusted 1 = 1140; n adjusted 2 = 705)	1.17 (0.69; 1.99), 0.562	1.33 (0.76; 2.33), 0.316	1.46 (0.74; 2.83), 0.283
***Alcohol exposure***			
*Antenatal* (n adjusted 1 = 993; n adjusted 2 = 650)	1.92 (0.93; 3.97), 0.080	**2.21 (1.04; 4.70), 0.040**	**2.93 (1.18; 7.23), 0.020**
*Postnatal– 10 weeks* (n adjusted 1 = 625; n adjusted 2 = 354)	0.79 (0.23; 2.67), 0.703	0.52 (0.14; 1.96), 0.332	0.69 (0.13; 3.75), 0.665
*Postnatal– 6 months* (n adjusted 1 = 571; n adjusted 2 = 323)	0.97 (0.37; 2.56), 0.949	1.07 (0.38; 2.97), 0.899	1.26 (0.34; 4.63), 0.733
*Postnatal– 12 months* (n adjusted 1 = 583; n adjusted 2 = 332)	0.82 (0.38; 1.79), 0.627	0.51 (0.20; 1.29), 0.157	0.73 (0.23; 2.35), 0.599
*Long-term exposure* (n adjusted 1 = 1140; n adjusted 2 = 705)	2.10 (0.83; 5.35), 0.119	2.32 (0.89; 6.05), 0.086	**3.74 (1.27; 11.03), 0.017**

IPV, Intimate partner violence

^a^ Adjusted based on directed acyclic graph (DAG)–adjusted for antenatal maternal psychosocial risk factor (in postnatal models); sex; recruitment site; HIV exposure; maternal education achievement; SES quartile

^b^ Adjusted based on DAG with additional variables–adjusted for antenatal maternal psychosocial risk factor (in postnatal models); sex; recruitment site; HIV exposure; maternal education achievement; SES quartile; maternal urine cotinine (smoke exposure); PM10; weight for age z-score at birth; duration of breastfeeding; season of birth

*Still significant if 1% significance level considered

**Table 6:** Logistic regression—Severe/hospitalized LRTI vs maternal psychosocial risk factors stratified by age.

	0–3 months^a^	3–6 months^b^	6–9 months^c^	9–12 months^d^
Maternal psychosocial risk factor	Adjusted OR (95% CI), p-value	Adjusted OR (95% CI), p-value	Adjusted OR (95% CI), p-value	Adjusted OR (95% CI), p-value
***Depression***				
*Antenatal* (n 1 = 994; n 2 = 936; n 3 = 994; n 4 = 994)	1.23 (0.74; 2.07), 0.425	**2.55 (1.08; 6.01), 0.032**	0.84 (0.28; 2.50), 0.760	1.16 (0.50; 2.69), 0.731
*Postnatal—10 weeks* (n 1 = 627; n 2 = 538; n 3 = 627; n 4 = 627)	1.29 (0.60; 2.79), 0.520	1.00 (0.24; 4.16), 0.996	1.99 (0.55; 7.14), 0.291	0.95 (0.30; 3.07), 0.936
*Postnatal—6 months* (n 1 = 579; n 2 = 546; n 3 = 579; n 4 = 536)	1.39 (0.62; 3.12), 0.431	0.93 (0.23; 3.76), 0.921	1.83 (0.42; 8.01), 0.424	0.30 (0.04; 2.46), 0.260
*Postnatal—12 months* (n 1 = 653; n 2 = 624; n 3 = 624; n 4 = 653)	**2.46 (1.24; 4.87), 0.010**	0.80 (0.21; 3.14), 0.752	1.80 (0.54; 5.97), 0.335	1.53 (0.51; 4.61), 0.448
***Psychological distress***				
*Antenatal* (n 1 = 991; n 2 = 933; n 3 = 991; n 4 = 991)	1.44 (0.85; 2.45), 0.177	1.11 (0.42; 2.94), 0.829	1.22 (0.42; 3.54), 0.719	1.53 (0.66; 3.57), 0.325
*Postnatal—10 weeks* (n 1 = 628; n 2 = 539; n 3 = 628; n 4 = 628)	1.67 (0.68; 4.07), 0.260	3.33 (0.70; 15.93), 0.132	3.12 (0.79; 12.38), 0.106	1.12 (0.28; 4.52), 0.870
*Postnatal—6 month*s(n 1 = 578; n 2 = 546; n 3 = 578; n 4 = 535)	**3.01 (1.14; 7.96), 0.026**	0.74 (0.13; 4.20), 0.737	1.27 (0.19; 8.55), 0.807	0.45 (0.05; 4.31), 0.491
*Postnatal—12 months* (n 1 = 684; n 2 = 652; n 3 = 652; n 4 = 684)	1.66 (0.68; 4.06), 0.270	1.57 (0.36; 6.78), 0.547	**4.31 (1.04; 17.76), 0.043**	0.75 (0.15; 3.70), 0.727
***IPV***				
*Antenatal* (n 1 = 994; n 2 = 936; n 3 = 994; n 4 = 994)	0.92 (0.56; 1.51), 0.748	1.09 (0.46; 2.59), 0.851	1.04 (0.39; 2.78), 0.932	0.96 (0.43; 2.16), 0.920
*Postnatal—10 weeks* (n 1 = 628; n 2 = 539; n 3 = 628; n 4 = 628)	1.37 (0.69; 2.72), 0.367	**3.63 (1.06; 12.40), 0.040**	2.04 (0.58; 7.27), 0.269	0.82 (0.27; 2.45), 0.722
*Postnatal—6 months* (n 1 = 577; n 2 = 544; n 3 = 577; n 4 = 534)	**2.28 (1.14; 4.60), 0.021**	**3.54 (1.08; 11.53), 0.036**	0.63 (0.13; 3.04), 0.565	1.00 (0.31; 3.29), 0.997
*Postnatal—12 months* (n 1 = 675; n 2 = 644; n 3 = 644; n 4 = 675)	3.12 (1.71; 5.68), <0.001*	1.53 (0.52; 4.53), 0.440	1.04 (0.32; 3.36), 0.948	1.10 (0.44; 2.74), 0.846
***Alcohol exposure***				
*Antenatal* (n 1 = 993; n 2 = 935; n 3 = 993; n 4 = 993)	**2.95 (1.27; 6.84), 0.012**	1.60 (0.34; 7.55), 0.550	1.87 (0.39; 9.01), 0.435	**3.64 (1.10; 12.00), 0.034**
*Postnatal—10 weeks* (n 1 = 625; n 2 = 536 n 3 = 625; n 4 = 625)	0.91 (0.23; 3.56), 0.887	1.40 (0.14; 14.11), 0.774	1.15 (0.12; 11.36), 0.902	0.43 (0.04; 4.43), 0.476
*Postnatal—6 months* (n 1 = 571; n 2 = 538; n 3 = 571; n 4 = 528)	0.76 (0.17; 3.47), 0.723	2.31 (0.46; 11.65), 0.310	1.67 (0.19; 15.03), 0.648	0.61 (0.07; 5.32), 0.656
*Postnatal—12 months* (n 1 = 583; n 2 = 556; n 3 = 556; n 4 = 556)	0.46 (0.14; 1.52), 0.202	1.85 (0.46; 7.48), 0.390	0.46 (0.05; 4.15), 0.493	0.75 (0.18; 3.14), 0.689

IPV, Intimate partner violence.

^a-d^ Multiple logistic regression models adjusted for antenatal maternal psychosocial risk factor (in postnatal models); sex; recruitment site; HIV exposure; maternal education achievement; SES quartile; and LRTI in previous period

*Still significant if 1% significance level considered

#### Antenatal psychological distress

Exposure to antenatal maternal psychological distress was not associated with any LRTI or severe LRTI in the first year of life, Tables 3–6. However, in the stratified models that included all variables, the odds of an infant experiencing at least one LRTI episode between 3 and 6 months of age was 81% more likely in those whose mother suffered from antenatal psychological distress compared to those whose mothers did not adjusted OR = 1.81, 95% CI:1.07; 3.06, p-value: 0.026), S1 Table.

#### Antenatal IPV

Antenatal maternal IPV was not associated with any LRTI or severe LRTI Tables 1–6.

#### Antenatal alcohol consumption

Antenatal maternal alcohol consumption had a 3-fold increased odds with at least one infant LRTI episode in the first 3 months of life compared to those whose mothers did not consume alcohol on a regular basis (adjusted OR = 2.90, 95% CI: 1.40; 6.03, p-value: 0.004), Table 4. In addition, antenatal maternal alcohol consumption was associated with any severe/ hospitalised LRTI episode in both adjusted models (adjusted OR_a_ = 2.21, 95% CI: 1.04; 4.70, p-value: 0.040; adjusted OR_b_ = 2.93, 95% CI: 1.18; 7.23, p-value: 0.020) respectively, Table 5. In the stratified analyses, antenatal maternal alcohol consumption was associated with severe LRTI in the first 3 months of age (adjusted OR = 2.95, 95% CI: 1.27; 6.84, p-value = 0.012), and LRTI between 9 and 12 months of age (adjusted OR = 3.64, 95% CI: 1.10; 12.00, p-value: 0.034), Table 6.

### Postnatal maternal psychosocial risk factor associations with LRTI

#### Postnatal maternal depression

Postnatal maternal depressive symptoms measured at a 12-month visit was not associated with LRTI when the minimum set of confounder variables were included. However, there was an association with any LRTI episode in the first year of life when additional variables, including mediator variables such as maternal smoking, were included in the model (adjusted OR_b_ = 2.06, 95% CI: 1.08; 3.96, p-value: 0.029), Table 3. In the stratified analyses, LRTI in the first 3 months of life was most strongly associated with postnatal maternal depression measured at 12 months (adjusted OR = 1.97 95 CI%: 1.11; 3.47, p-value: 0.020). Similarly, postnatal maternal depressive symptoms measured at 12 months were strongly associated with severe LRTI in the first three months of age.

#### Postnatal maternal psychological distress

In the stratified analyses, postnatal maternal psychological distress measured at 6-months was associated with both LRTI (adjusted OR = 2.50, 95% CI: 1.11; 5.61, p-value: 0.026) and severe LRTI (adjusted OR = 3.01, 95% CI: 1.14; 7.96, p-value: 0.026) in the 3 months of age. In addition, postnatal maternal psychological distress measured at the 12-months was associated with severe LRTI between 6 and 9 months of age (adjusted OR = 4.31, 95% CI: 1.04; 17.76, p-value: 0.043). Further, in the stratified analyses which included all variables (confounders and mediators), postnatal maternal psychological distress measured at 10-weeks was strongly associated with LRTI between 3–6 months and severe LRTI between the ages of 3–6 and 6–9 months, S1 and S2 Tables.

#### Postnatal maternal IPV

Postnatal maternal IPV was strongly associated with any LRTI in the first year of life in the multiple logistic regression model that adjusted for confounding variables found by the DAG (adjusted OR_a_ = 1.50, 95% CI: 1.03; 2.18, p-value: 0.032), Table 3; however this association fell away when including additional covariates such as infant smoke exposure, indoor air pollution exposure, weight-for-age Z-score at birth, duration of exclusive breastfeeding and season of birth. In the stratified analyses maternal IPV exposure measured at the 6-month and 12-month scheduled visits were found to be associated with LRTI between 0–3 months (adjust OR = 2.89, 95% CI: 1.62; 5.16, p-value: <0.001; adjusted OR = 2.13, 95% CI: 1.30; 3.48, p-value: 0.003). Similarly, postnatal IPV was strongly associated with any severe LRTI episode in the first year of life in both multiple regression models (adjusted OR_a_ = 2.02, 95% CI: 1.25; 3.28, p-value: 0.004; adjusted OR_b_ = 1.93, 95% CI: 1.00; 3.70, p-value = 0.049), Table 5. In addition, maternal IPV exposure measured at the 10-week and the 6-months visit increased the odds of infant severe/hospitalised LRTI between 3–6 months of age 3.6-fold and 3.5-fold, respectively, relative to those not exposed.

#### Postnatal maternal alcohol consumption

Postnatal maternal alcohol use was not associated with any LRTI outcome.

### Associations between long-term maternal psychosocial risk factor exposure and LRTI outcomes

#### Long-term maternal depression exposure

Long-term maternal depression was not significantly associated with any LRTI or severe/hospitalised LRTI in the first year of life.

#### Long-term maternal psychological distress exposure

Long-term exposure to maternal psychological distress had a strong association with severe/hospitalised LRTI in the first year of life and was significant at both 1% and 5% significant levels. A child whose mother who suffered from both long-term maternal psychological distress, had a 2.6-fold increased odds of at least one severe/hospitalised LRTI episode in the fully adjusted model compared to those whose mother were not exposed, (adjusted OR_1_ = 2.60, 95% CI: 1.45; 4.65, p-value: 0.001), Table 6.

#### Long-term maternal IPV exposure

Long-term IPV exposure was not associated with LRTI outcomes.

#### Long-term maternal alcohol consumption

Long-term maternal alcohol use was not associated with any LRTI or severe LRTI.

## Discussion

In this study, approximately one-third of the infants had an episode of LRTI, and 31% these episodes were severe or hospitalised events, despite high immunisation coverage, adequate nutrition and almost no child HIV infection. High prevalence of maternal psychosocial risk factors was also observed, with one-third of the women exposed to antenatal IPV, and one in five women suffering from antenatal depression and/or psychological distress. Notably, several maternal psychosocial risk factors, including depression, psychological distress, alcohol consumption and IPV were associated with LRTI in infants. These findings identify maternal psychosocial risk factors as overlooked risk factors for early childhood LRTI. Our data suggest that women should be screened and treated for maternal psychosocial risk factors (eg depression, IPV, alcohol use) during pregnancy and the post-partum period.

In a UK-based study, researchers found an association between maternal perinatal depression and 27% increased risk of one or more LRTI episode in early childhood [19]. Likewise, our study found an association between maternal depression and LRTI, as well as severe or hospitalised LRTI. Antenatal maternal depression was associated with severe LRTI episodes in early life, while postnatal maternal depression was also associated with early LRTI events. Similarly, our study found long-term maternal psychological distress was highly associated with severe LRTI. Thus, a combination of depression, anxiety and distress over longer periods of time may have a greater impact on child health. In addition, postnatal maternal psychological distress measured at 10-weeks was strongly linked with LRTI and severe LRTI after three months of age, even when potential mediators such as maternal smoking and breast feeding were included in the analysis. This suggests that maternal psychological distress may have an effect on infant LRTI that is not solely explained by maternal smoking/ breast feeding. Further, antenatal maternal alcohol consumption was also associated with severe or hospitalised LRTI. The dangers of alcohol consumption during pregnancy on foetal development are well-known; the DCHS has previously reported that antenatal maternal alcohol abuse was a determinant of impaired lung function in infants at 6 weeks of age [18]. This could predispose children to respiratory illnesses, such as LRTI, in the first year of life. However, increased alcohol consumption could also be prompted by the presence of other psychosocial risk factors, such as depression, psychological distress or IPV exposure; thus, high alcohol use during pregnancy may mediate these relationships.

In a LMIC context, research conducted in Bangladesh, India and Nepal found an association between severe postpartum domestic abuse in mothers and high incidence rates of illness, including acute respiratory tract infection, in their infants [21, 22]. In this study, postnatal maternal IPV exposure proved to be consistently associated with infant LRTI, including severe forms of respiratory illness. Although it is difficult to make causal inferences about the relationship of IPV exposure with child health outcomes, it is notable that IPV measured at 10-weeks was found to be associated with severe LRTI at 3 to 6 months of age. A range of mechanisms may underly this association, including altered childcare practices. Regardless of the precise underlying mechanisms, the link between postnatal maternal IPV exposure and severe infant LRTI is concerning given the high incidence of early childhood LRTI and prevalence of IPV in South Africa and other LMICs.

The current study findings are consistent with a prior analysis from the DCHS study that investigated the associations between maternal psychosocial risk factors and early child wheezing [17]. In that analysis, postnatal maternal psychological distress and IPV were associated with the occurrence and recurrence of child wheeze, an association that again was not solely explained by potential mediators such as maternal smoking [17, 43]. These consistent findings suggest that maternal psychosocial risk factors are strongly associated with early childhood respiratory illnesses.

Antenatal maternal psychosocial risk factors have been widely considered to impact foetal growth, through biological mechanisms, such as the release of maternal stress hormones. This may later impact physical growth and development, including lung development, thereby predisposing infants to respiratory illnesses [6, 44, 45]. Stress hormones, such as cortisol, which are heightened in a mother suffering from psychosocial risk factors (particularly antenatal psychological distress), may have in utero effects, disturbing the infant’s hypothalamic-pituitary-adrenal (HPA) axis [46]. In turn, increased infant cortisol levels could affect the infant’s stress response, consequently suppressing the infant’s immune system [46].

An impaired maternal-child relationship has been proposed as an explanation for the association between postnatal maternal IPV (and other psychosocial risk factors) exposure and child respiratory illnesses [47, 48]. Maternal exposure to IPV, depression or distress may disrupt a mother’s ability to provide the appropriate care for her child [20, 49, 50]. The infants’ HPA axis could also be impacted postnatally through the mother-child interaction [13, 46]. A disrupted interaction between the mother (or primary caregiver) and the child has been found to negatively impact a child’s self-regulation ability, including stress regulation [13, 46], making the infant more susceptible to LRTI or severe LRTI. This may also be the case with long-term exposure to maternal psychosocial risk factors, as the current study observed a greater increased risk of infant LRTI when the mother suffered from long-term psychological distress. Nevertheless, reverse causation cannot be ruled out, as a mother may suffer from depressive or anxiety symptoms if their child is ill, and the symptoms may vary depending on the severity of the child’s illness.

A strength of this study is the longitudinal and prospective measurement of multiple risk factors, including clinical, sociodemographic and maternal psychosocial; given the paucity of similar data in the region and LMIC settings. The strong active surveillance of LRTI outcomes, allowed us to accurately record LRTI episodes and the severity of the episodes. In addition, this analysis included a large population-based sample, and these results are likely to be generalisable to many communities in Sub-Saharan Africa and other LMICs.

Several limitations deserve discussion. The first, a cross-sectional design does not allow ascription of causation or temporality. However, we addressed this by considering a stratified analysis based on age to determine where postnatal maternal psychosocial risk factors had the strongest associations with LRTI events during the first year of life. Second, there is the possibility that unmeasured confounders may have contributed to our results. However, we adjusted for major risk factors known to be associated with LRTI. Third, loss to follow-up of participants may also have an impact on the findings. Although, we have high cohort retention. Fourth, we used maternal self-report to determine psychosocial illness, and participants may downplay symptoms for a range of reasons. However, the study staff have established a close relationship with mother-infant pairs and all questionnaires were completed in a private room to ensure confidentiality. Furthermore, confirmation of psychosocial risk factors was established by means of structured diagnostic interviews within DCHS [24].

## Conclusion

In conclusion, our findings suggest that maternal psychosocial risk factors (such as antenatal and postnatal maternal depression, postnatal psychological distress, postnatal IPV, and antenatal alcohol use) are highly associated with LRTI in infancy in a LMIC setting. These associations held even when potential mediators such as maternal smoking were included in the analyses. This is particularly concerning given that psychosocial risk factors are often overlooked aspects of both maternal and child health. The associations found here highlight the need for better screening for maternal psychosocial risk factors, and for interventions to reduce such exposures. Such interventions may not only improve maternal psychosocial health, but may also reduce the burden of infant LRTI, a leading cause of childhood mortality and morbidity worldwide.

## Supporting information

S1 Table Logistic regression—LRTI ever vs maternal psychosocial risk factors stratified by age. (DOCX)

S2 Table Logistic regression—Severe/hospitalized LRTI vs maternal psychosocial risk factors stratified by age. (DOCX)

## References

[1] Global, regional, and national age-sex specific mortality for 264 causes of death, 1980–2016: a systematic analysis for the Global Burden of Disease Study 2016. Lancet. 2016: 10.1016/S0140-6736(17)32152-9. PMC5605883 28919116

[2] Zar HJ, Ferkol TW. The global burden of respiratory disease-impact on child health. Pediatr Pulmonol. 2014;49(5):430–4. Epub 2014/03/13. 10.1002/ppul.23030 . 24610581

[3] Zar H, Barnett W, Myer L, Stein D, Nicol M. Investigating the early-life determinants of illness in Africa: the Drakenstein Child Health Study. Thorax. 2015;70(6):592–4. 10.1136/thoraxjnl-2014-206242 25228292 PMC5107608

[4] Dagvadorj A, Ota E, Shahrook S, Baljinnyam Olkhanud P, Takehara K, Hikita N, et al Hospitalization risk factors for children’s lower respiratory tract infection: A population-based, cross-sectional study in Mongolia. Sci Rep. 2016;6:24615 Epub 2016/04/20. 10.1038/srep24615 27090182 PMC4835771

[5] Reyes M, Perzanowski MS, Whyatt RM, Kelvin EA, Rundle AG, Diaz DM, et al Relationship between maternal demoralization, wheeze, and immunoglobulin E among inner-city children. Ann Allergy Asthma Immunol. 2011;107(1):42–9 e1. Epub 2011/06/28. 10.1016/j.anai.2011.03.004 21704884 PMC3135280

[6] Ramratnam SK, Visness CM, Jaffee KF, Bloomberg GR, Kattan M, Sandel MT, et al Relationships among Maternal Stress and Depression, Type 2 Responses, and Recurrent Wheezing at Age 3 Years in Low-Income Urban Families. Am J Respir Crit Care Med. 2017;195(5):674–81. Epub 2016/09/23. 10.1164/rccm.201602-0272OC 27654103 PMC5363974

[7] Mathilda Chiu YH, Coull BA, Cohen S, Wooley A, Wright RJ. Prenatal and postnatal maternal stress and wheeze in urban children: effect of maternal sensitization. Am J Respir Crit Care Med. 2012;186(2):147–54. Epub 2012/05/15. 10.1164/rccm.201201-0162OC 22582161 PMC3406080

[8] Klinnert MD, Nelson HS, Price MR, Adinoff AD, Leung DY, Mrazek DA. Onset and persistence of childhood asthma: predictors from infancy. Pediatrics. 2001;108(4):E69 Epub 2001/10/03. 10.1542/peds.108.4.e69 . 11581477

[9] Kozyrskyj AL, Letourneau NL, Kang LJ, Salmani M. Associations between postpartum depressive symptoms and childhood asthma diminish with child age. Clin Exp Allergy. 2017;47(3):324–30. Epub 2016/10/23. 10.1111/cea.12837 . 27770463

[10] Kozyrskyj AL, Mai XM, McGrath P, Hayglass KT, Becker AB, Macneil B. Continued exposure to maternal distress in early life is associated with an increased risk of childhood asthma. Am J Respir Crit Care Med. 2008;177(2):142–7. Epub 2007/10/13. 10.1164/rccm.200703-381OC . 17932381

[11] Lange NE, Bunyavanich S, Silberg JL, Canino G, Rosner BA, Celedon JC. Parental psychosocial stress and asthma morbidity in Puerto Rican twins. J Allergy Clin Immunol. 2011;127(3):734–40 e1-7. Epub 2011/01/05. 10.1016/j.jaci.2010.11.010 21194742 PMC3057225

[12] Giallo R, Bahreinian S, Brown S, Cooklin A, Kingston D, Kozyrskyj A. Maternal depressive symptoms across early childhood and asthma in school children: findings from a Longitudinal Australian Population Based Study. PLoS One. 2015;10(3):e0121459 Epub 2015/03/27. 10.1371/journal.pone.0121459 25811851 PMC4374762

[13] Wright RJ. Prenatal maternal stress and early caregiving experiences: implications for childhood asthma risk. Paediatr Perinat Epidemiol. 2007;21 Suppl 3:8–14. Epub 2007/11/21. 10.1111/j.1365-3016.2007.00879.x . 17935570

[14] Cheng TS, Chen H, Lee T, Teoh OH, Shek LP, Lee BW, et al An independent association of prenatal depression with wheezing and anxiety with rhinitis in infancy. Pediatr Allergy Immunol. 2015;26(8):765–71. Epub 2015/08/04. 10.1111/pai.12453 . 26235785

[15] Flanigan C, Sheikh A, DunnGalvin A, Brew BK, Almqvist C, Nwaru BI. Prenatal maternal psychosocial stress and offspring’s asthma and allergic disease: A systematic review and meta-analysis. Clin Exp Allergy. 2018;48(4):403–14. Epub 2018/01/14. 10.1111/cea.13091 . 29331049

[16] Andersson NW, Hansen MV, Larsen AD, Hougaard KS, Kolstad HA, Schlunssen V. Prenatal maternal stress and atopic diseases in the child: a systematic review of observational human studies. Allergy. 2016;71(1):15–26. Epub 2015/09/24. 10.1111/all.12762 26395995 PMC5054838

[17] MacGinty RP, Lesosky M, Barnett W, Stein DJ, Zar HJ. Associations between maternal mental health and early child wheezing in a South African birth cohort. Pediatr Pulmonol. 2018;53(6):741–54. Epub 2018/04/11. 10.1002/ppul.24008 29635887 PMC6001799

[18] Gray D, Willemse L, Visagie A, Czovek D, Nduru P, Vanker A, et al Determinants of early-life lung function in African infants. Thorax. 2017;72(5):445–50. Epub 2016/11/20. 10.1136/thoraxjnl-2015-207401 27856821 PMC5520243

[19] Ban L, Gibson JE, West J, Tata LJ. Association between perinatal depression in mothers and the risk of childhood infections in offspring: a population-based cohort study. BMC Public Health. 2010;10:799 Epub 2011/01/05. 10.1186/1471-2458-10-799 21194453 PMC3022867

[20] Asling-Monemi K, Naved RT, Persson LA. Violence against women and increases in the risk of diarrheal disease and respiratory tract infections in infancy: a prospective cohort study in Bangladesh. Arch Pediatr Adolesc Med. 2009;163(10):931–6. Epub 2009/10/07. 10.1001/archpediatrics.2009.167 . 19805712

[21] Silverman JG, Decker MR, Gupta J, Kapur N, Raj A, Naved RT. Maternal experiences of intimate partner violence and child morbidity in Bangladesh: evidence from a national Bangladeshi sample. Arch Pediatr Adolesc Med. 2009;163(8):700–5. Epub 2009/08/05. 10.1001/archpediatrics.2009.115 19652100 PMC4456175

[22] Ferdousy EZ, Matin MA. Association between intimate partner violence and child morbidity in South Asia. J Health Popul Nutr. 2015;33:16 Epub 2016/01/31. 10.1186/s41043-015-0016-y 26825360 PMC5025981

[23] Zar HJ, Barnett W, Myer L, Nicol MP. Childhood pneumonia—the Drakenstein Child Health Study. S Afr Med J. 2016;106(7):642–3. Epub 2016/07/08. 10.7196/SAMJ.2016.v106i7.11108 . 27384352

[24] Stein D, Koen N, Donald K, Adnams C, Koopowitz S, Lund C, et al Investigating the psychosocial determinants of child health in Africa: The Drakenstein Child Health Study. J Neurosci Methods. 2015;252:27–35. Epub 2015/03/24. 10.1016/j.jneumeth.2015.03.016 25797842 PMC4556362

[25] Brittain K, Myer L, Koen N, Koopowitz S, Donald KA, Barnett W, et al Risk Factors for Antenatal Depression and Associations with Infant Birth Outcomes: Results From a South African Birth Cohort Study. Paediatric and Perinatal Epidemiology. 2015;29(6):505–14. 10.1111/ppe.12216 26236987

[26] Barnett W, Brittain K, Sorsdahl K, Zar HJ, Stein DJ. Maternal participant experience in a South African birth cohort study enrolling healthy pregnant women and their infants. Philos Ethics Humanit Med. 2016;11(1):3 Epub 2016/07/21. 10.1186/s13010-016-0036-2 27435596 PMC4952056

[27] le Roux DM, Myer L, Nicol MP, Zar HJ. Incidence of childhood pneumonia: facility-based surveillance estimate compared to measured incidence in a South African birth cohort study. BMJ Open. 2015;5(12):e009111 Epub 2015/12/20. 10.1136/bmjopen-2015-009111 26685027 PMC4691755

[28] Integrated management of child illness. Distance learning course: chart booklet. In: Organization WH, editor. World Health Organization2014.

[29] Cox JL, Holden JM, Sagovsky R. Detection of postnatal depression: Development of the 10-item Edinburgh Postnatal Depression Scale. Br J Psychiatry. 1987;150:782–6. 10.1192/bjp.150.6.782 3651732

[30] Hartley M, Tomlinson M, Greco E, Comulada WS, Stewart J, le Roux I, et al Depressed mood in pregnancy: prevalence and correlates in two Cape Town peri-urban settlements. Reprod Health. 2011;8:9 Epub 2011/05/04. 10.1186/1742-4755-8-9 21535876 PMC3113332

[31] Beusenberg M, Orley J. A user’s guide to the self reporting questionnaire (SRQ). Geneva: World Health Organization 1994.

[32] Rumble S, Swartz L, Parry C, Zwarenstein M. Prevalence of psychiatric morbidity in the adult population of a rural South African village. Psychol Med. 1996;26:997–1007. 10.1017/S0033291700035327 8878332

[33] Harpham T, Reichenheim M, Oser R, Thomas E, Hamid N, Jaswal S, et al Measuring mental health in a cost-effective manner. Health Policy Plan. 2003;18(3):344–9. 10.1093/heapol/czg041 12917276

[34] Ventevogel P, De Vries G, Scholte W, Shinwari N, Faiz H, Nassery R, et al Properties of the Hopkins Symptom Checklist-25 (HSCL-25) and the Self-Reporting Questionnaire (SRQ-20) as screening instruments used in primary care in Afghanistan. Soc Psychiatry Psychiatr Epidemiol. 2007;42 (4):328–35. 10.1007/s00127-007-0161-8 17370049

[35] Jewkes R. Intimate partner violence: causes and prevention. The Lancet. 2002;359(9315):1423–9. 10.1016/s0140-6736(02)08357-5. 11978358

[36] Shamu S, Abrahams N, Temmerman M, Musekiwa A, Zarowsky C. A systematic review of African studies on intimate partner violence against pregnant women: prevalence and risk factors. PloS one. 2011;6(3):e17591 10.1371/journal.pone.0017591 21408120 PMC3050907

[37] WHO ASSIST Working Group. The Alcohol, Smoking and Substance Involvement Screening Test (ASSIST): development, reliability and feasibility. 2002 [cited 97 9]. 1183–94]. 10.1046/j.1360-0443.2002.00185.x 12199834

[38] Vanker A, Barnett W, Workman L, Nduru PM, Sly PD, Gie RP, et al Early-life exposure to indoor air pollution or tobacco smoke and lower respiratory tract illness and wheezing in African infants: a longitudinal birth cohort study. The Lancet Planetary Health. 2017;1(8):e328–e36. 10.1016/s2542-5196(17)30134-1 29167839 PMC5681433

[39] Government Gazette. Republic of South Africa Department of Environmental Affairs. National Ambient Air Quality Standards. 2009.

[40] Budree S, Stein DJ, Brittain K, Goddard E, Koen N, Barnett W, et al Maternal and infant factors had a significant impact on birthweight and longitudinal growth in a South African birth cohort. Acta Paediatr. 2017;106(11):1793–801. Epub 2017/08/11. 10.1111/apa.14015 28796908 PMC5656834

[41] Fenton T, Kim J. A systematic review and meta-analysis to revise the Fenton growth chart for preterm infants. BMC Pediatr. 2013;13:59 10.1186/1471-2431-13-59 23601190 PMC3637477

[42] Myer L, Stein D, Grimsrud A, Seedat S, Williams D. Social determinants of psychological distress in a nationally-representative sample of South African adults. Soc Sci Med. 2008;66:1828–40. 10.1016/j.socscimed.2008.01.025 18299167 PMC3203636

[43] le Roux DM, Nicol MP, Myer L, Vanker A, Stadler JAM, von Delft E, et al Lower Respiratory Tract Infections in Children in a Well-vaccinated South African Birth Cohort: Spectrum of Disease and Risk Factors. Clin Infect Dis. 2019 Epub 2019/03/30. 10.1093/cid/ciz017 . 30925191

[44] Wright RJ, Cohen S, Carey V, Weiss ST, Gold DR. Parental stress as a predictor of wheezing in infancy: a prospective birth-cohort study. Am J Respir Crit Care Med. 2002;165(3):358–65. Epub 2002/01/31. 10.1164/ajrccm.165.3.2102016 . 11818321

[45] McLean DE, Hatfield-Timajchy K, Wingo PA, Floyd RL. Psychosocial Measurement: Implications for the Study of Preterm Delivery in Black Women. American Journal of Preventive Medicine. 1993;9(6):39–81. 10.1016/s0749-3797(18)30665-2 8123286

[46] Wright RJ. Epidemiology of stress and asthma: from constricting communities and fragile families to epigenetics. Immunol Allergy Clin North Am. 2011;31(1):19–39. Epub 2010/11/26. 10.1016/j.iac.2010.09.011 21094921 PMC3052958

[47] Suglia SF, Enlow MB, Kullowatz A, Wright RJ. Maternal intimate partner violence and increased asthma incidence in children: buffering effects of supportive caregiving. Arch Pediatr Adolesc Med. 2009;163(3):244–50. Epub 2009/03/04. 10.1001/archpediatrics.2008.555 19255392 PMC3094096

[48] Bair-Merritt MH, Voegtline K, Ghazarian SR, Granger DA, Blair C, Family Life Project I, et al Maternal intimate partner violence exposure, child cortisol reactivity and child asthma. Child Abuse Negl. 2015;48:50–7. Epub 2014/12/02. 10.1016/j.chiabu.2014.11.003 25435104 PMC4446253

[49] Hassan BK, Werneck GL, Hasselmann MH. Maternal mental health and nutritional status of six-month-old infants. Rev Saude Publica. 2016;50:7 Epub 2016/03/24. 10.1590/S1518-8787.2016050006237 27007683 PMC4794770

[50] Nwabuzor Ogbonnaya I, Keeney AJ, Villodas MT. The role of co-occurring intimate partner violence, alcohol use, drug use, and depressive symptoms on disciplinary practices of mothers involved with child welfare. Child Abuse Negl. 2019;90:76–87. Epub 2019/02/16. 10.1016/j.chiabu.2019.02.002 . 30769190

