# Supplementary material for: Maternal psychosocial risk factors and lower respiratory tract infection (LRTI) during infancy in a South African birth cohort
Source: PLoS One. 2019 Dec 30;14(12):e0226144. doi: 10.1371/journal.pone.0226144 (PMC6936815; doi:10.1371/journal.pone.0226144)
Supplement: S1 Table — (DOCX) [file pone.0226144.s001.docx]

**S1 Table: Logistic regression - LRTI ever vs maternal psychosocial risk factors stratified by age**

|  | **0-3 months^a^** | **3-6 months^b^** | **6-9 months^c^** | **9-12 months^d^** |
| --- | --- | --- | --- | --- |
| **Maternal psychosocial risk factor** | **Adjusted OR (95%CI), p-value** | **Adjusted OR (95%CI), p-value** | **Adjusted OR (95%CI), p-value** | **Adjusted OR (95%CI), p-value** |
| ***Depression*** |  |  |  |  |
| *Antenatal* (n 1=678; n 2=678; n 3=678; n 4=678) | 0.86 (0.49; 1.51), 0.607 | 1.21 (0.72; 2.02), 0.476 | 0.98 (0.53; 1.84), 0.959 | 0.56 (0.28; 1.14), 0.109 |
| *Postnatal - 10 weeks* (n 1=446; n 2=416; n 3=446; n 4=446) | 1.46 (0.670; 3.04), 0.316 | 0.84 (0.36; 1.95), 0.682 | 1.26 (0.54; 2.94), 0.586 | 1.04 (0.40; 2.72), 0.939 |
| *Postnatal - 6 months* (n 1=413; n 2=386; n 3=413; n 4=413) | 1.57 (0.76; 3.23), 0.219 | 0.54 (0.22; 1.32), 0.178 | 1.23 (0.48; 3.14), 0.673 | 0.76 (0.27; 2.11), 0.600 |
| *Postnatal - 12 months* (n 1=467; n 2=467; n 3=467; n 4=467) | **2.88 (1.43; 5.77), 0.003^*^** | 1.36 (0.67; 2.74), 0.394 | 0.80 (0.33; 1.92), 0.620 | 0.97 (0.41; 2.30), 0.951 |
| ***Psychological distress*** |  |  |  |  |
| *Antenatal* (n 1=677; n 2= 677; n 3=677; n 4=677) | 1.23 (0.70; 2.17), 0.468 | **1.81 (1.07; 3.06), 0.026** | 1.22 (0.64; 2.35), 0.545 | 1.06 (0.54; 2.09), 0.858 |
| *Postnatal - 10 weeks* (n 1=447; n 2=417; n 3=447; n 4=447) | **2.47 (1.033; 5.93), 0.042** | 1.45 (0.51; 4.12), 0.483 | **3.21 (1.21; 8.49), 0.019** | 0.38 (0.07; 1.65), 0.176 |
| *Postnatal - 6 months* (n 1=413; n 2=387; n 3=413; n 4=413) | 2.47 (0.95; 6.43), 0.063 | **0.19 (0.05; 0.69), 0.012** | 0.78 (0.21; 2.86), 0.709 | 1.06 (0.30; 3.75), 0.927 |
| *Postnatal - 12 months* (n 1=486; n 2=486; n 3=486; n 4=486) | 2.21 (0.93; 5.21), 0.071 | 0.85 (0.37; 1.99), 0.716 | 1.65 (0.65; 4.19), 0.288 | 0.64 (0.20; 2.11), 0.465 |
| ***IPV^5^*** |  |  |  |  |
| *Antenatal* (n 1=678; n 2=678; n 3=678; n 4=678) | 0.76 (0.45; 1.29), 0.309 | 1.47 (0.91; 2.39), 0.114 | 1.08 (0.60; 1.96), 0.789 | 1.08 (0.60; 1.93), 0.808 |
| *Postnatal - 10 weeks* (n 1=443; n 2=417; n 3=447; n 4=447) | 1.38 (0.71; 2.69), 0.339 | 1.45 (0.71; 2.94), 0.309 | 0.78 (0.33; 1.83), 0.563 | 1.31 (0.57; 3.00), 0.523 |
| *Postnatal - 6 months* (n 1=412; n 2=385; n 3=412; n 4=412) | **2.79 (1.40; 5.57), 0.004*** | 1.09 (0.53; 2.23), 0.821 | 1.05 (0.44; 2.51), 0.915 | 1.49 (0.68; 3.30), 0.321 |
| *Postnatal - 12 months* (n 1=483; n 2=483; n 3=461; n 4=483) | **2.36 (1.28; 4.35), 0.006*** | 0.65 (0.35; 1.20), 0.166 | 0.70 (0.33; 1.46), 0.339 | 1.39 (0.70; 2.75), 0.346 |
| ***Alcohol exposure*** |  |  |  |  |
| *Antenatal* (n 1=679; n 2=677; n 3 =677; n 4=677) | **3.65 (1.57; 8.48), 0.003*** | 0.52 (0.17; 1.54), 0.237 | 2.26 (0.87; 5.83), 0.093 | 1.34 (0.41; 4.41), 0.633 |
| *Postnatal - 10 weeks* (n 1=444; n 2=414; n 3=444; n 4=444) | 0.54 (0.12; 2.47), 0.427 | 0.71 (0.14; 3.57), 0.680 | 2.93 (0.79; 10.91), 0.110 | 0.54 (0.08; 3.43), 0.510 |
| *Postnatal - 6 months* (n 1=410; n 2=383; n 3=410; n 4=410) | 1.29 (0.37; 4.51), 0.685 | 1.69 (0.54; 5.27), 0.363 | 0.90 (0.20; 3.96), 0.887 | 0.67 (0.13; 3.41), 0.634 |
| *Postnatal - 12 months* (n 1=417; n 2=417; n 3=417; n 4=417) | 2.01 (0.80; 5.05), 0.138 | 1.77 (0.76; 4.12), 0.186 | **0.19 (0.05; 0.73), 0.016** | 1.05 (0.33; 3.38), 0.930 |

IPV, Intimate partner violence.

^a-d^ Multiple logistic regression models adjusted for antenatal maternal psychosocial risk factor (in postnatal models); sex; recruitment site; HIV exposure; maternal education achievement; SES quartile; maternal urine cotinine (smoke exposure); PM10; weight for age z-score at birth; duration of breastfeeding; season of birth; and LRTI in previous period

* Still significant if 1% significance level considered
