# Supplementary material for: Maternal psychosocial risk factors and lower respiratory tract infection (LRTI) during infancy in a South African birth cohort
Source: PLoS One. 2019 Dec 30;14(12):e0226144. doi: 10.1371/journal.pone.0226144 (PMC6936815; doi:10.1371/journal.pone.0226144)
Supplement: S2 Table — (DOCX) [file pone.0226144.s002.docx]

**S2 Table: Logistic regression - Severe/hospitalized LRTI vs maternal psychosocial risk factors stratified by age**

|  | **0-3 months^a^** | **3-6 months^b^** | **6-9 months^c^** | **9-12 months^d^** |
| --- | --- | --- | --- | --- |
| **Maternal psychosocial risk factor** | **Adjusted OR (95% CI), p-value** | **Adjusted OR (95% CI), p-value** | **Adjusted OR (95% CI), p-value** | **Adjusted OR (95% CI), p-value** |
| ***Depression*** |  |  |  |  |
| *Antenatal* (n 1=678; n 2=637; n 3=518; n 4=637) | 0.89 (0.45; 1.74), 0.727 | 1.69 (0.56; 5.12), 0.355 | 1.01 (0.27; 3.86), 0.984 | 1.63 (0.58; 4.56), 0.355 |
| *Postnatal - 10 weeks* (n 1=446; n 2=378; n 3=341; n 4=416) | 1.64 (0.64; 4.18), 0.301 | 2.02 (0.40; 10.28), 0.397 | 2.40 (0.46; 12.63), 0.300 | 1.15 (0.27; 4.95), 0.847 |
| *Postnatal - 6 months* (n 1=413; n 2=292; n 3=259; n 4=247) | 1.40 (0.57; 3.46), 0.463 | 0.91 (0.15; 5.63), 0.916 | 1.74 (0.30; 10.11), 0.539 | 0.29 (0.02; 3.68), 0.343 |
| *Postnatal - 12 months* (n 1=469; n 2=445; n 3=361; n 4=445) | **3.53 (1.46; 8.53), 0.005^*^** | 0.72 (0.13; 3.97), 0.706 | 1.23 (0.24; 6.21), 0.805 | 1.07 (0.27; 4.32), 0.920 |
| ***Psychological distress*** |  |  |  |  |
| *Antenatal* (n 1=677; n 2=636; n 3=517; n 4=636) | 1.49 (0.77; 2.92), 0.239 | 1.29 (0.38; 4.34), 0.684 | 1.16 (0.29; 4.55), 0.833 | 1.76 (0.60; 5.19), 0.304 |
| *Postnatal - 10 weeks* (n 1=447; n 2=379; n 3=341; n 4=417) | 2.60 (0.88; 7.71), 0.086 | **18.64 (2.15; 161.35), 0.008^*^** | **6.41 (1.12; 36.88), 0.037** | 0.42 (0.05; 3.55), 0.428 |
| *Postnatal - 6 months* (n 1=413; n 2=260; n 3=259; n 4=248) | 2.69 (0.85; 8.49), 0.091 | - | 2.14 (0.19; 23.64), 0.533 | 0.70 (0.06; 8.14), 0.774 |
| *Postnatal - 12 months* (n 1=486; n 2=463; n 3=376; n 4=463) | 2.52 (0.88; 7.18), 0.084 | 2.37 (0.49; 11.58), 0.286 | 2.06 (0.34; 12.48), 0.431 | 0.41 (0.06; 3.04), 0.383 |
| ***IPV*** |  |  |  |  |
| *Antenatal* (n 1=678; n 2=637; n 3=579; n 4=637) | 0.76 (0.40; 1.47), 0.419 | 0.76 (0.24; 2.38), 0.632 | 1.69 (0.48; 5.94), 0.409 | 1.16 (0.43; 3.17), 0.769 |
| *Postnatal - 10 weeks* (n 1=447; n 2=379; n 3=342; n 4=417) | 1.66 (0.70; 3.92), 0.252 | **6.39 (1.23; 33.21), 0.027** | 1.12 (0.19; 6.54), 0.901 | 0.68 (0.17; 2.71), 0.586 |
| *Postnatal - 6 months* (n 1=412; n 2=291; n 3=259; n 4=247) | 1.87 (0.82; 4.31), 0.139 | 4.36 (0.83; 23.03), 0.083 | 0.88 (0.12; 6.38), 0.900 | 1.51 (0.28; 8.17), 0.634 |
| *Postnatal - 12 months* (n 1=483; n 2=461; n 3=376; n 4=461) | **3.15 (1.42; 7.00), 0.005^*^** | 1.22 (0.32; 4.72), 0.771 | 0.52 (0.11; 2.54), 0.419 | 1.11 (0.33; 3.69), 0.866 |
| ***Alcohol exposure*** |  |  |  |  |
| *Antenatal* (n 1=679; n 2=636; n 3=518; n 4=636) | **4.63 (1.77; 12.06), 0.002^*^** | 1.53 (0.26; 9.02), 0.640 | 1.36 (0.22; 8.35), 0.738 | **5.68 (1.33; 24.25), 0.019** |
| *Postnatal - 10 weeks* (n 1=444; n 2=376 n 3=339; n 4=414) | 1.38 (0.25; 7.62), 0.709 | 2.33 (0.18; 30.40), 0.519 | 0.97 (0.06; 14.55), 0.981 | 0.10 (0.00; 2.42), 0.159 |
| *Postnatal - 6 months* (n 1=410; n 2=290; n 3=256; n 4=245) | 0.48 (0.06; 4.16), 0.506 | 6.49 (0.78; 53.81), 0.083 | 2.50 (0.19; 33.16), 0.487 | 0.66 (0.04; 10.92), 0.775 |
| *Postnatal - 12 months* (n 1=417; n 2=396; n 3=280; n 4=396) | 0.50 (0.10; 2.42), 0.390 | 2.67 (0.51; 13.91), 0.244 | 1 - | 1.50 (0.28; 7.93), 0.636 |

IPV, Intimate partner violence.

^a-d^ Multiple logistic regression models adjusted for antenatal maternal psychosocial risk factor (in postnatal models); sex; recruitment site; HIV exposure; maternal education achievement; SES quartile; maternal urine cotinine (smoke exposure); PM10; weight for age z-score at birth; duration of breastfeeding; season of birth; and LRTI in previous period

*Still significant if 1% significance level considered
